# An Analysis on How Socioeconomic and Geographical Factors Influence Recognition of Sport Related Concussion in the Lone Star State

**DOI:** 10.1177/30502225251403608

**Published:** 2025-12-17

**Authors:** Sarah Bivans, Joshua A. Beitchman, Cason Hicks, Christine Woods, C. Munro Cullum, Mathew Stokes

**Affiliations:** 1 University of Texas Southwestern Medical Center, Dallas, TX, USA; 2 Texas Scottish Rite Hospital for Children, Dallas, TX, USA

**Keywords:** sport related concussion, high-school, athlete, pediatric, socioeconomic status

## Abstract

**Objective:**

To describe how sociodemographic factors may impact the reported distribution of Sport related concussion (SRC) in high-school sports.

**Introduction:**

SRC may affect athletes’ daily functioning and ability perform academically. Consideration of sociodemographic factors in the reporting of SRC are needed to address possible inequities in concussion diagnosis.

**Methods:**

This review of data from the ConTex2 database identified 6377 cases of SRC among high-school athletes who were then analyzed by geographic location and socioeconomic status.

**Results:**

Reports of SRC by geographic location revealed cities had a smaller percentage of total SRC than suburbs in swimming/diving, water polo, wrestling, and marching band. Lower SES schools had a smaller percentage of reported SRC in cheerleading and marching band. Similarly, lower incidence rates of SRC were found in schools in cities and of lower SES.

**Conclusion:**

Consideration of sociodemographic factors in SRC may provide more equitable allocation of resources and improve concussion care for all student athletes.

## Introduction

High school athletes who experience sport-related concussion (SRC) are at increased risk for injury-induced symptoms that can affect their ability to perform at school and in their daily lives. Current estimates suggest that at least 15% of the 2.5 million American high school athletes will experience at least 1 concussion related to playing a sport or being physically active, and many studies have highlighted the concern for underreporting among this population.^1,2^ While most athletes improve over the first few weeks following injury, up to 30% report lingering symptoms including cognitive, somatic, headache, vestibular, ocular, affective, and sleep impairments.^3-7^

Certain patient-specific factors such as age, medical/psychological history, sex, prior concussions, and resilience may help to predict those who will develop lingering symptoms, but these factors cannot identify individual risk.^8-14^ Adolescent studies have identified how sociodemographic factors can influence health outcomes; however, limited studies have identified how these factors might influence recognition, incidence, and knowledge of SRC.^15-21^ Available resources between urban, suburban, and rural schools fundamentally differ, which may result in disparities when it comes to available protective equipment, concussion education, and medical personnel.^18,19^ Prior studies have shown how having athletic trainers (AT) on teams increases concussion diagnosis rates as well as education regarding concussion identification and prevention.^18,22^ However, their availability depends on school district and team funding, as lower socioeconomic status (SES) teams are less likely to have an AT on staff.^23^ Amongst rural populations, athletes initially present in emergency departments rather than outpatient or specialist centers for concussion care, reinforcing their limited access to specialized concussion care.^22^ Together, these studies suggest that athletes in lower socioeconomic areas may be at increased risk for underreported brain injuries. These are only some of the disparities student athletes face across our communities, yet each of these complex socioeconomic factors offers an opportunity to intervene in order to improve healthcare for youth athletes.

Texas has more student athletes than any other state, with an estimated 827,446 students that attend private and public school participating in sports in the 2022 to 2023 school year.^24^ Despite these numbers, there is a lack of studies looking at the incidence of pediatric SRC across the U.S., including Texas, specifically in regard to demographic factors. The Rank One™ – Concussion-Texas (ConTex) database, referred to as ConTex2, was created as a quality improvement project between concussion researchers at The University of Texas Southwestern Medical Center, the Medical Advisory Committee of the University Interscholastic League (UIL), and Rank One™ to help address these gaps. Using the expanded ConTex2 database, this study aims to determine groups that may be at risk for under-recognition of SRC and guide future analysis that investigates reporting patterns across demographics. These findings can help advocate for equal access to appropriate resources for all students.

## Methods

### Study Population

This retrospective review of prospectively collected data included 6377 participants entered into the ConTex2 database between August 2021 and May 2023. Participants were included if they were diagnosed with a known or suspected SRC and enrolled in ninth through 12th grade at the time of injury. The overall ConTex2 database contains de-identified information regarding pediatric athletic injuries. All Texas schools participating in the UIL are encouraged to report concussion data to the ConTex2 data portal; as of 2019, the UIL mandated concussion reporting for all 6A UIL-participating sports. To be classified as 6A in Texas, a school must meet an enrollment threshold of 2275 students or more. Sport related concussion was defined as an event that occurred during an official game or practice and was suspected or diagnosed by a healthcare provider.^25^ Following a reported diagnosis of SRC, data was gathered from the student’s records including the participants age, gender, grade, sport, mechanism of the injury, school, and school district. Injuries that occurred outside of an athletic practice or competition and injuries with insufficient data to determine how or where the injury was sustained were excluded (n = 1315). This was a quality improvement project between Rank One™, the UIL, and The University of Texas Southwestern Medical Center which required a data use agreement to allow for the transmission of data and received ethical approval from IRB.

### Procedures and Measures

#### Inclusion Criteria

Participants and their respective sports were described within a calendar school year (August 1st-July 31st). Sports were included in the final data analysis if there were 10 or more total concussions for the respective school year (2021-2022) and (2022-2023; Table 2). UIL Texas considers marching band a sport and was therefore included in the final analysis. A total of 13 sports for the 2021 to 2022 school year and 16 sports for the 2022 to 2023 school year were included. For sports in which only 1 year met inclusion criteria, the total represents data from both academic years. Data for the 2022 to 2023 school year represents injuries through May of 2023 as data collection was incomplete at the time of the study.

#### Geographic Classification

Geographic analysis involved classifying each high school attended by participants as city, urban, rural, or town as defined by the National Center for Education Statistics (NCES). The NCES classifies campuses into categories based on population size and proximity to urban areas.^26^ Further descriptions of the NCES classifications can be found in the supplemental A. For data analysis purposes rural and town classifications were combined under the rural title as there were too few participants who attended schools in the town classification to properly analyze these as separate entities.

#### Socioeconomic Classification

In order to provide an estimate of socioeconomic status, the percentage of students eligible to receive free or reduced-price lunch at each high school attended by study participants was used.^19,27^ Free and reduced-price lunch eligibility is determined by household income relative to the federal poverty guidelines according to the USDA.^28,29^ The percentage of students at each school who met these requirements was then used to classify each participant into 1 of 3 categories based on their school’s characteristics: low (free and reduced lunch rate > 66%), average (free and reduced lunch rate 33%-66%), or high (free and reduced lunch rate < 33%) socioeconomic status. Private schools did not report a percentage of students that received free or reduced-price lunch and therefore those students were classified under the high SES classification.

#### Incidence

Traditionally, research articles on sport related concussion utilize an injury rate [number of injuries/number of athlete exposures (practice or game)] to characterize the burden of injury.^30^ Given that these data were collected from the ConTex2 database which is a statewide mandatory reporting system for 6A schools, it encompasses over 200 schools across multiple school districts, each with a different number of games, and practices per week. While the use of a large, mandatory reporting system like ConTex2 strengthens the population-based nature of the analysis, the variability in school schedules necessitated the use of a surrogate marker to calculate incidence, as athlete exposure data were not available. Incidence in this study was defined as total number of injuries per 1000 students. School level data on the number of students enrolled at each school for the corresponding academic school year was obtained from the Public Education Information Management System (PEIMS) Standard Reports generated by the Texas Education Agency.^31^

#### Mapping

A sub-group analysis was performed on the Dallas-Fort-Worth (DFW) area. DFW is home to a large diverse population and trends seen here may be extrapolated to other large metropolitan areas. The following counties were included as part of the DFW area: Denton, Collin, Dallas, Rockwall, and Tarrant. Incidence rates were calculated per school district in the DFW area as described above to facilitate mapping. SRC sustained by students attending private schools were mapped based on physical location of the school. A map of incidence rates per district across the DFW area was creating using a Geographic information system (GIS; ESRI, (2011). ArcGIS: Release 10). See Supplemental A for further methods of map design and calculations.

#### Statistical Analysis

Sample means and standard deviations were used to describe continuous variables and frequencies with percentages were used to characterize categorical variables. Pearson’s Chi square analyses were conducted for each sport to analyze differences in the distribution of injuries across geographic and socioeconomic classifications. When the Chi square analyses were significant, post hoc pair-wise comparisons were conducted using Fisher’s exact test between groups.^32^ For post hoc comparisons, the Bonferroni correction was applied. All statistical analyses were carried out using GraphPad Prism 9.5.1 (GraphPad Software, San Diego, California USA). The level of significance was set at *p* ≤ .05 for primary analyses.

## Results

### Sample Demographics

Participant demographics are summarized in Table 1. Overall, there were 6377 student athletes at 289 unique schools who reportedly had a known or suspected SRC over the 2021 to 2023 study period [Male n = 3620 (56.77%), Female n = 2757 (43.23%), Table 1]. Throughout 2021 to 2022, 3619 athletes [Male n = 2137 (59.05%), Female n = 1482 (40.95%)] with SRC were reported amongst a total student body enrollment of 528 494 students. For the 2022 to 2023 year, 2744 injuries [Male n = 1482 (54.01%), Female n = 1262 (45.99%)] were reported among a total study body enrollment of 617 389 students. Mean age at time of injury was 16.2 years (Male = 16.23 ± 1.18, Female = 16.19 ± 1.09). Athletes of lower socioeconomic status with reported SRC were older than those of higher SES (Low = 16.28 ± 1.19, High = 16.17 ± 1.13, *P* < .05). Sports that met the inclusion criteria are shown in Tables 2 and 3. For the entire study period, football accounted for the most reported SRC of any sport, with 2386 injuries (37.42%) followed by soccer n = 1199(18.8%), basketball n = 757(11.87%), and wrestling n = 456(7.15%).

**Table 1.** Demographics of Participants Based on Their School’s NCES Geographic Classification and Socioeconomic Classification for the Entire Study Period (2021-2023).

	Geographic location				Socioeconomic status				
	City	Suburb	Rural	*P*-value	High	Average	Low	*P*-value	Total
Schools n (%)	133 (46.02)	118 (40.83)	38 (13.15)		115 (39.79)	91 (31.49)	83 (28.72)		289
Number of Students enrolled n (%)									
2021-2022 n (%)	216 204 (40.91)	237 148 (44.87)	75 142 (14.22)		142 444 (26.95)	194 787 (36.86)	191 263 (36.19)		528 494
2022-2023 n (%)	253 444 (41.05)	281 444 (45.59)	82 501 (13.36)		188 141 (30.47)	230 741 (37.37)	198 507 (32.16)		617 389
Concussions overall n (%)	2489 (39.03)	2917 (45.74)	971 (15.23)		2204 (34.56)	2436 (38.20)	1737 (27.24)		6377
2021-2022 n (%)	1386 (38.30)^b^	1635 (45.18)^a^	598 (16.52)^a,b^	<.05	1175 (32.47)^a,b^	1426 (39.40)^a,c^	1018 (28.13)^b,c^	<.05	3619
2022-2023 n (%)	1100 (40.09)	1272 (46.35)	372 (13.56)	.589	1025 (37.35)^a,b^	1003 (36.55)^a,c^	716 (26.10)^b,c^	<.05	2744
Male n (%)	1442 (39.83)	1621 (44.78)	557 (15.39)	.199	1307 (36.11)^a^	1316 (36.35)^a^	997 (27.54)	<.05	3620 (56.77)
Female n (%)	1047 (37.98)	1296 (47.01)	414 (15.02)		897 (32.54)	1120 (40.62)	740 (26.84)		2757 (43.23)
Average Age (years)	16.23 ± 1.12	16.22 ± 1.16	16.17 ± 1.16	.274	16.17 ± 1.13	16.20 ± 1.12	16.28 ± 1.19	<.05	16.21 ± 1.14

Abbreviation: NCES, National Center for Education Statistics.

a,b,c Represents pairs of individual groupings that were significantly different (*P*≤0.01) based upon post hoc analysis and Bonferroni correction. ^b,c^Used to denote if additional pairs of groupings were significant. Superscripts are used to make comparisons only across individual rows and either geographic or socioeconomic classifications.

**Table 2.** Number of Concussions Based on a Participant’s School’s NCES Geographic Classification. The First Set of Columns Represents the Combined Analysis of the Entire Study Period (2021-2023); Subsequent Columns Represent the 2021/2022 and 2022/2023 Academic Years, Respectively.

	Combined 2021-2023					2021-2022					2022-2023				
Sport	City n (%)	Suburb n (%)	Rural n (%)	Total n (%)	*P*-value	City n (%)	Suburb n (%)	Rural n (%)	Total n (%)	*P*-value	City n (%)	Suburb n (%)	Rural n (%)	Total n (%)	*P*-value
Band	3 (0.12)^a^	17 (0.58)^a^	2 (0.21)	22 (0.34)	<.05						0 (0.00)^a^	12 (0.94)^a^	1 (0.27)	13 (0.47)	<.05
Baseball	63 (2.53)	90 (3.09)	33 (3.40)	186 (2.92)	.302	29 (2.09)	48 (2.94)	20 (3.34)	97 (2.68)	.196	34 (3.09)	42 (3.30)	13 (3.49)	89 (3.24)	.920
Basketball	271 (10.89)	362 (12.41)	124 (12.77)	757 (11.87)	.145	148 (10.68)	203 (12.42)	79 (13.21)	430 (11.88)	.185	123 (11.18)	159 (12.50)	45 (12.10)	327 (11.92)	.609
Cheerleading	91 (3.66)	109 (3.74)	49 (5.05)	249 (3.90)	.132	55 (3.97)	59 (3.61)	30 (5.02)	144 (3.98)	.321	36 (3.27)	50 (3.93)	19 (5.11)	105 (3.83)	.270
Cross Country	8 (0.32)	12 (0.41)	5 (0.51)	25 (0.39)	.697	2 (0.14)	8 (0.49)	3 (0.50)	13 (0.36)	N/A	6 (0.55)	4 (0.31)	2 (0.54)	12 (0.44)	N/A
Dance/Drill	8 (0.32)	12 (0.41)	8 (0.82)	28 (0.44)	.127	3 (0.22)	5 (0.31)	5 (0.84)	13 (0.36)	N/A	5 (0.45)	7 (0.55)	3 (0.81)	15 (0.55)	.728
Football	1011 (40.62)^a^	1009 (34.59)^a^	366 (37.69)	2386 (37.42)	<.05	603 (43.51)	650 (39.76)	242 (40.47)	1495 (41.31)	.102	408 (37.09)^a^	359 (28.22)^a^	124 (33.33)	891 (32.47)	<.05
Lacrosse	6 (0.24)	4 (0.14)	0 (0)	10 (0.16)	N/A						6 (0.55)	4 (0.31)	0 (0)	10 (0.36)	N/A
Soccer	481 (19.33)	562 (19.27)	156 (16.07)	1199 (18.80)	.060	262 (18.90)	297 (18.17)	99 (16.56)	658 (18.18)	.461	219 (19.91)	265 (20.83)	57 (15.32)	541 (19.72)	.062
Softball	175 (7.03)	164 (5.62)	61 (6.28)	400 (6.27)	.104	100 (7.22)	88 (5.38)	30 (5.02)	218 (6.02)	.057	75 (6.82)	76 (5.97)	31 (8.33)	182 (6.63)	.261
Swimming & Diving	15 (0.60)^a,b^	46 (1.58)^a^	16 (1.65)^b^	77 (1.21)	<.05	8 (0.58)^a^	27 (1.65)^a^	10 (1.67)	45 (1.24)	<.05	7 (0.64)	19 (1.49)	6 (1.61)	32 (1.17)	.105
Tennis	15 (0.60)	18 (0.62)	5 (0.51)	38 (0.60)	.936	10 (0.72)	7 (0.43)	4 (0.67)	21 (0.58)	.544	5 (0.45)	11 (0.86)	1 (0.27)	17 (0.62)	.291
Track & Field	41 (1.65)	44 (1.51)	16 (1.65)	101 (1.58)	.907	21 (1.52)	16 (0.98)	9 (1.51)	46 (1.27)	.362	20 (1.82)	28 (2.20)	7 (1.88)	55 (2.00)	.789
Volleyball	164 (6.59)	183 (6.27)	52 (5.36)	399 (6.26)	.404	92 (6.64)	113 (6.91)	34 (5.69)	239 (6.60)	.586	72 (6.55)	70 (5.50)	18 (4.84)	160 (5.83)	.379
Water Polo	9 (0.36)^a^	32 (1.10)^a^	3 (0.31)	44 (0.69)	<.05						9 (0.82)^a^	27 (2.12)^a^	3 (0.81)	39 (1.42)	<.05
Wrestling	128 (5.14)^a,b^	253 (8.67)^a^	75 (7.72)^b^	456 (7.15)	<.05	53 (3.82)^a^	114 (6.97)^a^	33 (5.52)	200 (5.53)	<.05	75 (6.82)^a,b^	139 (10.93)^a^	42 (11.29)^b^	256 (9.33)	<.05
Total	2489	2917	971	6377	.167	1386	1635	598	3619	.365	1100	1272	372	2744	.070

*P*-value for sports where there was an insufficient number of participants to analyze. Level of significance set at *p* ≤ .05 for Pearson’s Chi square analysis.

Abbreviations: N/A, not applicable; NCES, National Center for Education Statistics.

a,b Represents pairs of individual groupings where significance (*P*≤0.01) was seen with post hoc analysis and Bonferroni correction.

**Table 3.** Number of concussions based on a participant’s socioeconomic status classification. Socioeconomic classification was based upon the percentage of students eligible for free or reduced cost lunch at that participant’s school. The first set of columns represents the combined analysis of the entire study period (2021-2023); subsequent columns represent the 2021/2022 and 2022/2023 academic years, respectively.

	Combined 2021-2023					2021-2022					2022-2023				
Sport	High n (%)	Average n (%)	Low n (%)	Total n (%)	P Value	High n (%)	Average n (%)	Low n (%)	Total n (%)	P Value	High n (%)	Average n (%)	Low n (%)	Total n (%)	P Value
Band	8 (0.36)	13 (0.53)^a^	1 (0.06)^a^	22 (0.34)	<**.05**						4 (0.39)	9 (0.90)	0 (0.00)	13 (0.47)	N/A
Baseball	54 (2.45)	70 (2.87)	62 (3.57)	186 (2.92)	.115	25 (2.13)	39 (2.73)	33 (3.24)	97 (2.68)	.270	29 (2.83)	31 (3.09)	29 (4.05)	89 (3.24)	.346
Basketball	240 (10.89)	292 (11.99)	225 (12.95)	757 (11.87)	.135	126 (10.72)	164 (11.50)	140 (13.75)	430 (11.88)	.079	114 (11.12)	128 (12.76)	85 (11.87)	327 (11.92)	.522
Cheerleading	84 (3.81)	112 (4.60)^a^	53 (3.05)^a^	249 (3.90)	<**.05**	44 (3.74)	67 (4.70)	33 (3.24)	144 (3.98)	.170	40 (3.90)	45 (4.49)	20 (2.79)	105 (3.83)	.194
Cross Country	15 (0.68)	7 (0.29)	3 (0.17)	35 (0.39)	<**.05**	10 (0.85)	1 (0.07)	2 (0.20)	13 (0.36)	N/A	5 (0.49)	6 (0.60)	1 (0.14)	12 (0.44)	N/A
Dance/Drill	11 (0.50)	14 (0.57)	3 (0.17)	28 (0.44)	.134	7 (0.60)	5 (0.35)	1 (0.10)	13 (0.36)	N/A	4 (0.39)	9 (0.90)	2 (0.28)	15 (0.55)	.160
Football	851 (38.61)	878 (36.04)	657 (37.82)	2386 (37.42)	.180	490 (41.70)	588 (41.23)	417 (40.96)	1495 (41.31)	.938	361 (35.22)^a^	290 (28.91)^a^	240 (33.52)	891 (32.47)	<**.05**
Lacrosse	10 (0.45)	0 (0)	0 (0)	10 (0.16)	N/A						10 (0.98)	0 (0)	0 (0)	10 (0.36)	N/A
Soccer	407 (18.47)	463 (19.01)	329 (18.94)	1199 (18.80)	.882	212 (18.04)	256 (17.95)	190 (18.66)	658 (18.18)	.894	195 (19.02)	207 (20.64)	139 (19.41)	541 (19.72)	.641
Softball	114 (5.17)^a^	154 (6.32)	132 (7.60)^a^	400 (6.27)	<**.05**	61 (5.19)	89 (6.24)	68 (6.68)	218 (6.02)	.312	53 (5.17)^a^	65 (6.48)	64 (8.94)^a^	182 (6.63)	<**.05**
Swimming & Diving	28 (1.27)	32 (1.31)	17 (0.98)	77 (1.21)	.587	17 (1.45)	18 (1.26)	10 (0.98)	45 (1.24)	.617	11 (1.07)	14 (1.40)	7 (0.98)	32 (1.17)	.692
Tennis	14 (0.64)	13 (0.53)	11 (0.63)	38 (0.60)	.879	8 (0.68)	8 (0.56)	5 (0.49)	21 (0.58)	.837	6 (0.59)	5 (0.50)	6 (0.84)	17 (0.62)	.666
Track & Field	40 (1.81)	40 (1.64)	21 (1.21)	101 (1.58)	.305	19 (1.62)	18 (1.26)	9 (0.88)	46 (1.27)	.311	21 (2.05)	22 (2.19)	12 (1.68)	55 (2.00)	.746
Volleyball	125 (5.67)	177 (7.27)	97 (5.58)	399 (6.26)	<**.05**	71 (6.04)	109 (7.64)	59 (5.80)	239 (6.60)	.124	54 (5.27)	68 (6.78)	38 (5.31)	160 (5.83)	.274
Water Polo	19 (0.86)	12 (0.49)	13 (0.75)	44 (0.69)	.298						19 (1.85)	9 (0.90)	11 (1.54)	39 (1.42)	.183
Wrestling	184 (8.35)	159 (6.53)	113 (6.51)	456 (7.15)	<**.05**	85 (7.23)^a^	64 (4.49)^a^	51 (5.01)	200 (5.53)	<**.05**	99 (9.66)	95 (9.47)	62 (8.66)	256 (9.47)	.765
Total	2204	2436	1737	6377	.681	1175	1426	1018	3619	.761	1025	1003	716	2744	.651

*P*-value for sports where there were an insufficient number of participants to analyze. Level of significance set at *p* ≤ .05 for Pearson’s Chi square analysis.

Abbreviation: N/A, not applicable.

a Represents pairs of individual groupings that were significant (*P*≤0.01) with post hoc analysis and Bonferroni correction.

### Geographic Classification of SRC

Geographic classification was completed using NCES criteria of the school attended by the injured athlete. Overall number of reported concussions was lowest in rural areas and highest in the suburbs (city n = 2489, suburb n = 2917, or rural n = 971; Table 2). Chi square tests were then performed for each individual sport to determine if the distribution of reported SRC differed by geographic location. Sports with significant differences across geographic classifications include football (X^2^ = 20.88, *P* < .05), swimming and diving (X^2^ = 12.55, *P* < .05), water polo (X^2^ = 13.03, *P* < .05), wrestling (X^2^ = 25.78, *P* < .05), and marching band (X^2^ = 8.99, *P* < .05). Fisher exact post-hoc comparisons of individual groupings were then conducted to help provide direction on where these differences may exist; however, especially for sports with fewer participants, these data should be interpreted cautiously in the context of small sample sizes. The post hoc comparison revealed cities represented in ConTex2 may have a smaller percentage of overall reported concussions than suburbs in the following sports: swimming and diving [n = 15 (0.60%) vs n = 46 (1.58%), *P* < .01], water polo [n = 9 (0.36%) vs n = 32 (1.10%), *P* < .01], wrestling [n = 128 (5.14%) vs n = 253 (8.67%), *P* < .01], and marching band [n = 3 (0.12%) vs n = 17 (0.58%), *P* < .01]. Cities also had a smaller percentage of total reported SRC than rural areas in swimming and diving [n = 15 (0.60%) vs n = 16 (1.65%), *P* < .01] and wrestling [n = 128 (5.14%) vs n = 75 (7.72%), *P* < .01]. Cities had proportionally more concussions reported than suburbs in football [n = 1011 (40.62%) vs n = 1009 (34.59%), *P* < .01].

Individual school years showed similar findings as the combined analyses. For the 2021 to 2022 school year, swimming and diving (X^2^ = 8.12, *P* < .05), and wrestling (X^2^ = 14.24, *P* < .05) were the only sports with significant differences. Post hoc analysis revealed both sports had a smaller percentage of total concussions in the city classification when compared to suburbs: swimming and diving [n = 8 (0.58%) vs n = 27 (1.65%), *P* < .01] and wrestling [n = 53 (3.82%) vs n = 114 (6.97%), *P* < .01].

For the 2022 to 2023 school year, football (X^2^ = 21.30, *P* < .05), water polo (X^2^ = 8.33, *P* < .05), wrestling (X^2^ = 13.73, *P* < .05), and marching band (X^2^ = 11.52, *P* < .05) were found to have significant differences across geographic regions. Of these sports, cities had a smaller percentage of reported concussions than suburbs in water polo [n = 9 (0.82%) vs n = 27 (2.12%), *P* < .01], wrestling [n = 75 (6.82%) vs n = 139 (10.93%), *P* < .01], and marching band [n = 0 (0%) vs n = 12 (0.94%), *P* < .01]. Wrestling also has a smaller percentage of reported concussion in cities as compared to rural areas [n = 75 (6.82%) vs n = 42 (11.29%), *P* < .01]. Conversely, football was again noted to comprise a larger percentage of total concussions in the city classification than in suburbs [n = 408 (37.09%) vs n = 359 (28.22%), *P* < .01].

### Socioeconomic Classification of SRC

Socioeconomic classification was completed using the percentage of students eligible for free or reduced-price meals at the school in which the injured athlete attended. Despite having more students enrolled than schools of high SES, schools of lower SES had less reported concussions overall: high (n = 2204), average (n = 2436), or low (n = 1737). Individual sports with significant differences across SES included cheerleading (X^2^ = 6.54, *P* < .05), cross country (X^2^ = 7.52, *P* < .05), softball (X^2^ = 9.75, *P* < .05), volleyball (X^2^ = 6.86, *P* < .05), wrestling (X^2^ = 7.28, *P* < .05), and marching band (X^2^ = 6.72, *P* < .05). Post-hoc analysis of individual groupings revealed that schools of lower SES had a smaller percentage of total reported concussions than schools of average socioeconomic status in the following sports: cheerleading [n = 53 (3.05%) vs n = 112 (4.60%), *P* < .01] and marching band [n = 1 (0.06%) vs n = 13 (0.53%), *P* < .01]. Conversely, softball comprised a larger percentage of the overall concussions in schools of low socioeconomic status compared to those of high socioeconomic status [n = 132 (7.6%) vs n = 114 (5.17%), *P* < .01]. Fewer concussions were reported in schools of lower SES compared to high SES in cross country [n = 3 (0.17%) vs n = 15 (0.68%)], volleyball [n = 97 (5.58%) vs n = 125 (5.67%)], and wrestling [n = 113 (6.51%) vs n = 184 (8.35%)]; however, post hoc analyses were not significant among these comparisons.

Analysis of individual school years revealed similar findings as the combined analysis. For the 2021 to 2022 school year, wrestling (X^2^ = 10.03, *P* < .05) was the only sport with significance. A smaller percentage of identified concussions were seen in wrestling at schools of average versus higher SES [n = 64 (4.49%) vs n = 85 (7.23%), *P* < .01]. For the 2022 to 2023 school year football (X^2^ = 9.68, *P* < .05) and softball (X^2^ = 9.72, *P* < .05) were the sports with significant differences across SES. Schools of average SES had a lower percentage of overall concussions reported in football than schools of higher SES [n = 290 (28.91%) vs n = 361 (35.22%), *P* < .01]. Softball again displayed the opposite relationship compared to most sports, with lower SES schools reporting a higher percentage of overall concussions compared to high SES schools [n = 64 (8.94%) vs n = 53 (5.17%), *P* < .01].

### Incidence of SRC

Incidence rates per 1000 students were calculated for each sport for individual academic years (Table 4). Total student enrollment was used as a surrogate, as athlete exposure (AE) data were not available. Given that not every student participates in athletics, observed incidence rates were expected to be lower than some previous published studies which range from 4.17 to 10.00 concussions per 10 000 athlete exposures.^33,34^ Overall incidence for the study period (2021-2023) was not calculated, as the number of students per school changes on a yearly basis.

**Table 4.** Incidence Rates by Sport. Rates were Calculated by Total Number of Concussions Per 1000 Students Enrolled at Schools for Each Respective Geographic and Socioeconomic Classification. Incidence Rates are Separated by Academic School Year.

	2021/2022 Academic year							2022/2023 Academic year						
	Geographic incidence			SES incidence				Geographic incidence			SES incidence			
Sport	City	Suburb	Rural	High	Average	Low	Total	City	Suburb	Rural	High	Average	Low	Total
Band								0.000	0.043	0.012	0.021	0.039	0.000	0.021
Baseball	0.134	0.202	0.266	0.176	0.200	0.173	0.184	0.134	0.149	0.158	0.154	0.134	0.146	0.144
Basketball	0.685	0.856	1.051	0.885	0.842	0.732	0.814	0.485	0.565	0.545	0.606	0.555	0.428	0.530
Cheerleading	0.254	0.249	0.399	0.309	0.344	0.173	0.272	0.142	0.178	0.230	0.213	0.195	0.101	0.170
Cross Country	0.009	0.034	0.040	0.070	0.005	0.010	0.025	0.024	0.014	0.024	0.027	0.026	0.005	0.019
Dance/Drill	0.014	0.021	0.067	0.049	0.026	0.005	0.025	0.020	0.025	0.036	0.021	0.039	0.010	0.024
Football	2.789	2.741	3.221	3.440	3.019	2.180	2.829	1.610	1.276	1.503	1.919	1.257	1.209	1.443
Lacrosse								0.024	0.014	0.000	0.053	0.000	0.000	0.016
Soccer	1.212	1.252	1.318	1.488	1.314	0.993	1.245	0.864	0.942	0.691	1.036	0.897	0.700	0.876
Softball	0.463	0.371	0.399	0.428	0.457	0.356	0.412	0.296	0.270	0.376	0.282	0.282	0.322	0.295
Swimming & Diving	0.037	0.114	0.133	0.119	0.092	0.052	0.085	0.028	0.068	0.073	0.058	0.061	0.035	0.052
Tennis	0.046	0.030	0.053	0.056	0.041	0.026	0.040	0.020	0.039	0.012	0.032	0.022	0.030	0.028
Track & Field	0.097	0.067	0.120	0.133	0.092	0.047	0.087	0.079	0.099	0.085	0.112	0.095	0.060	0.089
Volleyball	0.426	0.476	0.452	0.498	0.560	0.308	0.452	0.284	0.249	0.218	0.287	0.295	0.191	0.259
Water Polo								0.036	0.096	0.036	0.101	0.039	0.055	0.063
Wrestling	0.245	0.481	0.439	0.597	0.329	0.267	0.378	0.296	0.494	0.509	0.526	0.412	0.312	0.415
Total	6.411	6.894	7.958	8.249	7.321	5.323	6.848	4.340	4.520	4.509	5.448	4.347	3.607	4.445

For the 2021 to 2022 school year, the overall incidence of reported SRC was 6.85 concussions per 1000 students. Football experienced the highest incidence (2.83), followed by soccer (1.24) and basketball (0.81). Incidence was also calculated regarding geography and socioeconomic status. Rural areas had the highest incidence (7.96) compared to suburbs (6.89) and cities (6.41). In terms of socioeconomic status, schools of high SES had the highest incidence (8.25) compared to schools of average (7.32) and lower (5.32) SES.

Results of the 2022 to 2023 school year found lower incidence rates compared to the 2021 to 2022 school year. The overall incidence of SRC was 4.44 concussions per 1000 students. Football again demonstrated the highest incidence (1.44), followed by soccer (0.88), and basketball (0.52). In terms of geographic differences, the incidence was similar in suburban (4.52) and rural areas (4.51) compared to a lower incidence seen in cities (4.34). For SES, schools of higher SES had the highest incidence (5.45), followed by schools of average (4.35) and lower (3.61) SES.

### DFW Map of SRC Incidence

**Figure 1.**
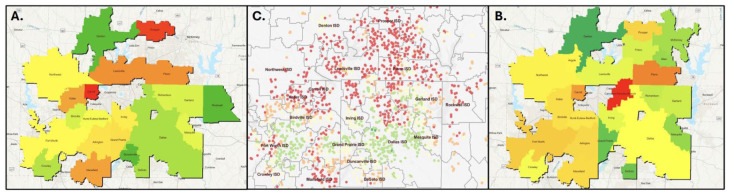
Map of incidence rates in the DFW area. (A) Map of incidence rates per district for the 2021 to 2022 school year. (B) Map of incidence rates per district for the 2022 to 2023 school year. (C) Income to poverty ratio of schools in the DFW area In maps A and B color coding by district with red representing the highest incidence rates, yellow average incidence, and green the lowest incidence. Map C Higher ratios are red with lower ratios represented by green.

A subgroup analysis was performed on the Dallas Fort Worth (DFW) area in order to identify local sociodemographic interactions in this large metroplex area (Figure 1). Incidence rates of overall concussions per 1000 students were calculated for each district. For the 2021 to 2022 school year, 1096 participants in 22 school districts in the DFW area were included. Incidence rates ranged widely from 0.35 to 19.37. Overall trends pointed to higher incidence in northern suburbs, with lower incidence rates in more southern urban areas. For the 2022 to 2023 school year, 768 participants in 25 school districts in the DFW area were included. Again, incidence ranged widely across districts (0.78-16.17). Similarly to the 2021 to 2022 school year data, higher incidence rates were seen in northern suburbs and in the western areas, while lower rates were seen toward the south.

## Discussion

High school athletes are at risk of sustaining sport related concussions in the United States.^35^ These injuries can result in students experiencing symptoms that can affect their ability to perform at school and in their daily lives. The State of Texas affords the largest number of high school athletes of any state and represents diverse geographic and SES communities. Our analyses provide a preliminary insight into how various factors may influence the recognition and reporting of SRC. Student athletes in Texas reported experiencing SRC across geographic and socioeconomic classifications, with the majority occurring amongst suburban communities and average socioeconomic status. Our data are consistent with previous reports that have found the highest number of adolescent SRCs occur in football, soccer, and basketball across all geographic and socioeconomic categories.^33,34,36-39^ Investigation into how SRC is distributed based on geographic factors revealed differences in certain sports between groupings. Sports in which these findings were statistically significant included football, swimming and diving, water polo, wrestling, and marching band. Post hoc analyses suggested that in some sports, including swimming and diving, water polo, wrestling, and marching band, proportionally fewer concussions were reported in cities compared to suburban areas. Conversely, football comprised a larger percentage of overall reported concussions in cities compared to suburban or rural areas. Further investigation into the relationship between socioeconomic status and SRC also revealed differences in reported concussions. Overall, lower SES schools had fewer reported concussions despite having higher enrollment numbers than schools of higher SES. Sports that had statistically significant differences across SES included cheerleading, cross country, softball, and marching band. In post hoc comparisons, cheerleading and marching band had a smaller percentage of overall reported concussions in lower SES schools compared to average or high SES schools. It should be noted that post hoc comparisons involve smaller subsets of the overall sample, which may limit statistical power; however, these comparisons serve to highlight potential areas of difference and direct future study in this area. Finally, a sub-group analysis in the DFW area found that a lower incidence of SRC was associated with lower SES areas. Together, these are some of the first findings in adolescent athletics that identify differences in reported SRC based on geography and SES. Consideration of these data may raise awareness amongst coaches, trainers, sideline providers, and clinicians to advocate for improved recognition of SRC amongst all athletes.

Some of the inherent differences across schools in different geographic areas include funding, ability to hire medical personnel, equip athletes, and provide concussion education.^18,23^ Our data provide evidence to suggest that the distribution of reported SRC across sports does, in fact, vary on the basis of geographic location. Preliminary analyses suggest that fewer concussions may be being reported in cities compared to suburbs in certain sports; however, further analysis is needed to replicate these results and delineate why this might occur. Incidence of SRC amongst geographic location for the 2021 to 2022 and 2022 to 2023 school years mirror these data, with cities reporting the lowest incidence when compared to suburban and rural areas. These trends are likely multifactorial but may be related to either true differences in incidence or more likely due to differences in reporting and possibly detection. Schools in cities may have fewer resources than their suburban counterparts, leading them to allocate the fewer resources they do have to sports such as football that pose the greatest risk of SRC. Additional factors that also impact these trends involve education and medical personnel availability. It has been previously demonstrated that athletes attending urban schools have less concussion knowledge, less frequently recognize the common signs and symptoms of concussion, and are less aware of the potential effects of multiple concussions.^18^ Thus, some urban high school athletes may be at a disadvantage when it comes to the timely recognition and diagnosis of SRC as they have less access to medical personnel, less concussion knowledge, and may be less likely to identify the symptoms of concussion on their own.

It is encouraging that there was a lack of differences in traditionally high-risk sports such as basketball, cheerleading, soccer, and volleyball. This may support the idea that schools are providing adequate resources to these higher-risk sports with the increase in concussion and health knowledge over the last few decades. Overall, it is understandable that schools have limited resources that must be appropriately distributed. However, efforts to also consider smaller, traditionally lower risk sports are needed to ensure concussions are being recognized and not underreported in these areas. Another aspect that may be contributing to these observed differences is the mechanism of concussion, which may vary based on geographic location. It has been previously demonstrated that children in rural locations are more likely to sustain concussions through motor vehicle accidents as compared to their urban counterparts.^40^ While this review did not detect any patterns in concussion when comparing rural and urban locations, these differences may be going unnoticed when only analyzing a specific mechanism of injury such as SRC. Future efforts to improve concussion detection, reporting, and education are needed across all geographic locations with a focus on urban athletes to improve concussion care for all high school athletes.

 Socioeconomic disparities are among the most fundamental causes of disparities in health equity; however, investigation using large-scale population-based studies evaluating the impact of SES on the incidence of SRC in high school athletes are limited. While some previous studies have explored the relationship between SES and SRC, the available data primarily focus on concussion knowledge, reporting attitudes, and access to medical personnel such as athletic trainers.^18,19,23^ One additional study found increased rates of concussion were correlated with higher household income; however, this was not specific to SRC per se.^41^ The results of the data presented here suggest that students attending schools with low socioeconomic status may have fewer concussions reported in certain sports compared to their peers that attend schools with higher SES. Furthermore, the overall incidence rates were lower in schools of lower SES when compared to those of average or high SES. These data are some of the first to analyze incidence rates in SRC in relation to SES and serve to raise awareness that differences may exist between these populations. While it is hypothesized that these differences may be due to differences in concussion reporting, further investigation is needed to explore this hypothesis and uncover additional potentially contributing factors. These trends are likely multifactorial but may be impacted by the fact that schools of lower SES have less access to athletic trainers, which could lead to underreporting and possibly underdiagnosis of SRC.^23,42^ Limited resources may prompt schools to prioritize athletic trainer attendance at football games and other high-impact sports, while smaller sports might not receive the same attention. Another factor that could potentially impact reporting is the availability of concussion education and knowledge. While is it is somewhat unclear the exact mechanisms that promote the inequity between health and SES, health literacy has been found to largely mediate the relationship.^43^ Disparities in health literacy in terms of concussion knowledge among school age athletes have been highlighted in previous studies.^18-20^ Additionally, those who attend schools with a greater proportion of low-income students may be less likely to have access to baseline neurocognitive testing, which may further impact their post concussive care.^44^ Together, these studies indicate how athletes in resource limited areas are potentially at greater risk for delayed recognition of SRC.

Across major cities in the United States there has been a trend of regional development in which suburban areas contain households with more resources and the means to have left the central city while those without those same resources often remain in inner urban areas.^45^ The Dallas-Fort Worth Metroplex is one of the most rapidly growing regions in the United States and is certainly no exception to this rule. Beginning as early as 1980, Dallas suburbs, which include mainly northern counties of Denton, Collin, and Rockwall began to experience rapid growth, far outpacing more urban regions in both Dallas and Tarrant counties (which contain the cities of Dallas and Fort Worth respectively).^45^ 2021 median household income data reflects these values as well with Dallas ($65,011) and Tarrant ($73,545) counties far below that of their suburban counterparts of Collin ($104,327), Denton ($96,265) and Rockwall ($111,595).^46^ Subgroup analysis of the DFW area allowed visualization of how geography and socioeconomic status may influence the distribution of reported SRC. Districts located in the lower income counties of Dallas and Tarrant such as Dallas ISD, Duncanville ISD, De Soto ISD, and Fort Worth ISD generally displayed lower incidence rates. This is in line with previous results in that lower income areas tend to have lower numbers of reported SRC, and it is possible that concussions may be going undiagnosed. Overall, mapping of the DFW area identified that areas with lower incidence of SRC reporting coincided with higher poverty rates. However, more data are needed to begin to understand how geography and socioeconomic status influence the rate of reporting for SRC across the state as well as nation-wide.

In terms of limitations, it should be noted that the data for the 2022 to 2023 school year was incomplete, making it difficult to make meaningful comparisons of trends over time. However, we can extrapolate that the 2022 to 2023 school year was on pace to have a similar number of concussions reported as the previous year with both years totaling an average of about 300 concussions per month. It is unclear why there was an increase in the number of schools that reported in 2022 to 2023; however, this could be attributed to increasing popularity of the Rank One™ program and statewide interest in concussion surveillance programs. Notably, an increase in number of schools reporting was not necessarily associated with an increase in the number of reported concussions overtime. Due to the unavailability of athlete exposure data, total student body enrollment was used as a surrogate marker to calculate incidence rates. Using total school population to calculate incidence may provide a limited analysis compared to studies that have data on athlete exposure for multiple reasons. First, not every student enrolled in a school plays sports and is at higher risk of experiencing a SRC. Additionally, there is potential for bias in the data given that it is hard to predict the magnitude of how school enrollment influences team sizes. However, it is known that larger schools tend to have larger rosters, and smaller schools often participate in sports with more limited active plays. One may also expect incidence rates to be biased to be lower in schools with larger enrollments. However, the results of this study sometimes found that incidence rates were lower even in schools with less students, for example when comparing schools of low and average SES. Although this approach lacks the granularity of exposure-based rates, it allowed a larger study with comparison across more diverse groups. Future population-based studies should aim to use athlete exposure data to ensure that this preliminary data can be replicated when controlling for this aspect. Since total student enrollment at each school was used to calculate incidence rates, this likely skewed the data for incidence in the 2022 to 2023 school year as we had complete data for student enrollment but not complete data for number of injuries. Another limitation of this study was the use of post hoc analyses to compare individual groupings. These preliminary analyses revealed that in many sports there were significant differences between categories, although pinpointing where these differences occur merits further investigation. Rather than serving as definitive conclusions, the post hoc analyses were aimed to guide future research that may be able to support and expand upon these preliminary observations. Another limitation of this data set was that race was not reported for the majority of athletes and therefore could not be analyzed. Race is often overlooked in in the SRC literature, and when possible, future studies should focus on how race/ethnicity factors may play into the differences in SRC detection and reporting across geographic regions.^47^ Those studies that do include race have found that there are disparities in how and where minoritized individuals receive post-concussion care and in the likelihood of reporting injuries.^48-50^ Another limitation is that differences in sex among geographic and socioeconomic classifications for individual sports was not analyzed due to limitations in sample size, even though sex differences have been reported in concussion risk and outcomes, thereby indicating a need for further investigation.^51^ Finally, the timing of this study during the COVID-19 pandemic may have affected school reporting.

## Conclusion

This report highlights important relationships between socioeconomic status and geographic location on the reporting of SRC in high school athletics in a U.S. state. In general, lower SES and location within urban cities was associated with fewer reported concussions. These differences were magnified in smaller sports, suggesting that funding issues and resource allocation may play a role in the reporting of SRC. Further population-based studies on the effect of sociodemographic factors on high school SRC are needed to replicate and extend these results. Systematic acquisition and review of such data will hopefully lead to improving the safety of youth sports across the country. In addition, concussion education should be provided in all settings, with a focus on making the information accessible and understandable to all learners.^52^ Future directions that could aid in increasing access to medical care in low resource settings include the use of telemedicine for concussion diagnosis and treatment as well as targeted education programs. Together, these interventions can help pave the way for a more equitable recognition of SRC for all student athletes, fostering a safer and more inclusive sports environment.

## Supplemental Material

sj-docx-1-gph-10.1177_30502225251403608 – Supplemental material for An Analysis on How Socioeconomic and Geographical Factors Influence Recognition of Sport Related Concussion in the Lone Star State Supplemental material, sj-docx-1-gph-10.1177_30502225251403608 for An Analysis on How Socioeconomic and Geographical Factors Influence Recognition of Sport Related Concussion in the Lone Star State by Sarah Bivans, Joshua A. Beitchman, Cason Hicks, Christine Woods, C. Munro Cullum and Mathew Stokes in Sage Open Pediatrics
