# Supplementary material for: An Analysis on How Socioeconomic and Geographical Factors Influence Recognition of Sport Related Concussion in the Lone Star State
Source: Sage Open Pediatr. 2025 Dec 17;12:30502225251403608. doi: 10.1177/30502225251403608 (PMC12712312; doi:10.1177/30502225251403608)
Supplement: sj-docx-1-gph-10.1177_30502225251403608 – Supplemental material for An Analysis on How Socioeconomic and Geographical Factors Influence Recognition of Sport Related Concussion in the Lone Star State [file sj-docx-1-gph-10.1177_30502225251403608.docx]

Supplemental

1. The NCES classifies campuses into categories based on population size and proximity to urban areas. NCES classifies campuses into twelve categories, composed of four basic types (City, Suburban, Town, and Rural). Descriptions of the four major categories are as follows: ‘City’ is defined as a territory inside an urbanized area and inside a principal city, ‘suburban’ as a territory outside a principal city and inside an urbanized area, ‘town’ as a territory inside an urban cluster, and ‘rural’ as a census defined rural territory. Urban areas that contain 50,000 or more people are designated as Urbanized Areas (UAs); urban areas that contain at least 2,500 and less than 50,000 people are designated as Urban Clusters (UCs). A core based statistical area (CBSA) is a geographic entity associated with at least one population core of 10,000 or more, plus adjacent territory that has a high degree of social and economic integration with the core, as measured by commuting ties. Principal Cities are incorporated places with a large population of residents and workers located within a CBSA. The term “rural” encompasses all population, housing, and territory not included within an urban area
2. A color-coding scale was created in Microsoft Excel and PowerPoint to represent the severity of incidence. The highest incidence was set as red and lowest as green, with yellow representing the average incidence. The hex color code was extracted and used to create the map using the GIS software. Separate maps were created for each school year. An additional map of individual schools in the DFW area was included for reference. Schools were color coded based on their income to poverty ratio (IPR). IPR compares family income to the federal poverty income threshold. For example, if family income is the same as the poverty income threshold (100%), then the IPR is 100. IPR ranges from 0 to 999. Red represents higher ratios and therefore higher income areas and green represents lower ratios.
